# Development of animal models for emerging infectious diseases by breaking the barrier of species susceptibility to human pathogens

**DOI:** 10.1080/22221751.2023.2178242

**Published:** 2023-02-23

**Authors:** Na Rong, Jiangning Liu

**Affiliations:** NHC Key Laboratory of Human Disease Comparative Medicine, Beijing Key Laboratory for Animal Models of Emerging and Remerging Infectious Diseases, Institute of Laboratory Animal Science, Chinese Academy of Medical Sciences and Comparative Medicine Center, Peking Union Medical College, Beijing, People’s Republic of China

**Keywords:** Emerging infectious diseases, animal model, susceptible animal, human pathogen, susceptibility spectrum

## Abstract

Outbreaks of emerging infectious diseases pose a serious threat to public health security, human health and economic development. After an outbreak, an animal model for an emerging infectious disease is urgently needed for studying the etiology, host immune mechanisms and pathology of the disease, evaluating the efficiency of vaccines or drugs against infection, and minimizing the time available for animal model development, which is usually hindered by the nonsusceptibility of common laboratory animals to human pathogens. Thus, we summarize the technologies and methods that induce animal susceptibility to human pathogens, which include viral receptor humanization, pathogen-targeted tissue humanization, immunodeficiency induction and screening for naturally susceptible animal species. Furthermore, the advantages and deficiencies of animal models developed using each method were analyzed, and these will guide the selection of susceptible animals and potentially reduce the time needed to develop animal models during epidemics.

## Introduction

Understanding the etiology, immunology and pathology of an infectious disease is fundamental for controlling pathogen transmission and developing vaccine- or drug-based strategies to prevent and cure the disease. Animal models, as patient surrogates, are needed to support this research. These models are developed by infecting susceptible laboratory animals with specific pathogens; in these animals, the process of pathogen entry and replication, host immune response, pathological injury and disease occurrence is reproduced. To facilitate the development and application of animal models for the study of infectious diseases, the functions of animal models as well as proposed solutions to resolve bottlenecks in the development of animal models are analyzed in the present review.

## Functions of animal models for infectious disease prevention and control

Using coronavirus disease 2019 (COVID-19) as an example, we will highlight the use of animal models to resolve key issues in the prevention and control of future infectious diseases. After the outbreak of COVID-19, animal models, including transgenic mice expressing human angiotensin-converting enzyme 2 (ACE2), rhesus monkeys and hamsters, were rapidly established [1–3]. Through animal experiments, SARS-CoV-2 was verified as the etiological pathogen of COVID-19, human ACE2 was confirmed as the entry receptor of SARS-CoV-2, and the characteristics of SARS-CoV-2 replication and the histological manifestations of COVID-19 were identified [2]. Using animal models, scientists found that SARS-CoV-2 is transmitted efficiently via direct contact, respiratory droplets and/or aerosols and that the conjunctiva is an alternative route for viral entry [4–6]. Furthermore, the specific immune response elicited by SARS-CoV-2 infection confers protection against secondary challenge with the same strain for a certain period, which guided the instilled confidence in serum therapy and vaccine development [7–8]. Moreover, animal models have been used to screen marketed drugs or novel antibodies and to identify effective candidates that can fulfil the emergency clinical demands [9–10]. In addition to drugs, a series of SARS-CoV-2 vaccine candidates developed using different technologies, including inactivated viruses, recombinant subunit proteins, adenovirus vectors, DNA and mRNA, have been evaluated in animal models and selected as potential candidates for clinical trials [11].

## Overcoming bottlenecks in the development of animal models for infectious diseases

An outbreak of a severe respiratory disease in early 2003 eventually led to more than 8000 cases and 774 deaths in 30 countries. Efforts were undertaken in several laboratories around the world to develop animal models, and several different animal models were ultimately developed (e.g. cynomolgus, African green and rhesus monkeys, ferrets and mice) [12]; however, studies on SARS-CoV were limited by animal model development for several months because it took a long time to screen the animals for susceptibility to SARS-CoV. The speed of animal model establishment partly limits emerging epidemic prevention and control because vaccine and drug development depend mainly on the timely and successful identification, screening and breeding of animals susceptible to the new pathogen.

Viruses are the main pathogens responsible for a series of emerging infectious diseases since the twenty-first century, e.g. SARS, influenza and avian influenza, hand, foot and mouth diseases, Ebola, Zika and Dengue. A property known as the specific host range of viruses means that most viruses are able to productively infect a set of host species, which varies substantially between different viruses [13]. However, some viruses have broad host ranges and infect multiple different host species, e.g. zoonotic diseases can be transmitted from animals to humans through direct contact or food, water and the environment. However, many viruses have a very limited host range and might only infect several or even a single host species. Poxviruses, which are responsible for several epidemics of lethal exanthematous diseases around the world in history, are considered emergent zoonotic viral diseases and exhibit the widest known host range [14]. In contrast to Poxviruses, hepatitis B virus (HBV) and human immunodeficiency virus (HIV) have extremely narrow host ranges; thus, animal models for HBV or AIDS infection and pathogenesis in humans have been lacking until now [15–16].

The fundamental factors that support the susceptibility of animals to human pathogens are establishing infection in host cells and breaking through the host immune defense system. A productive infection involves many viral replication events, including adhesion, entry, uncoating, replication, assembly and release. First, to establish an infection, the host cells must be susceptible and permissible [17–18]. A cell that expresses the viral receptor and allows adhesion and entry of the virus is thought to be susceptible to the virus [19]. However, the presence of a receptor is not sufficient to support viral replication. The permissibility of cells for viral replication is that the virus can successfully break the cellular defense mechanism consisting of a series of antiviral restriction factors, which impedes the critical steps of viral replication or triggers innate responses, and then replicate by efficiently using the metabolic machinery of the host. Most antiviral resistance factors are interferon-stimulated genes (ISGs), which are initiated by binding of interferon (IFN) to IFN receptors and then trigger a series of signalling cascades to protect against viral infection [20]. In addition to ISGs, some restriction factors block the key steps of virus replication. For example, the C-terminal domain of tripartite motif-containing protein 5 (TRIM5a) can bind retroviruses and subject the virus to the proteasome for degradation [21]. Bone marrow stromal cell antigen 2 (BST 2) has been identified as a restriction factor that prevents viral release, such as that of HIV-1 and human coronaviruses SARS-CoV-2, SARS-CoV and 229E, by tethering virions to the cell surface or intracellular membranes [22–25]. The susceptibility of animals to a specific human pathogen is involved in the susceptibility and permissibility of host cells and the immune defense system, all of which are mainly determined by the genetic and immune status diversity between different animal species.

**Figure 1.**
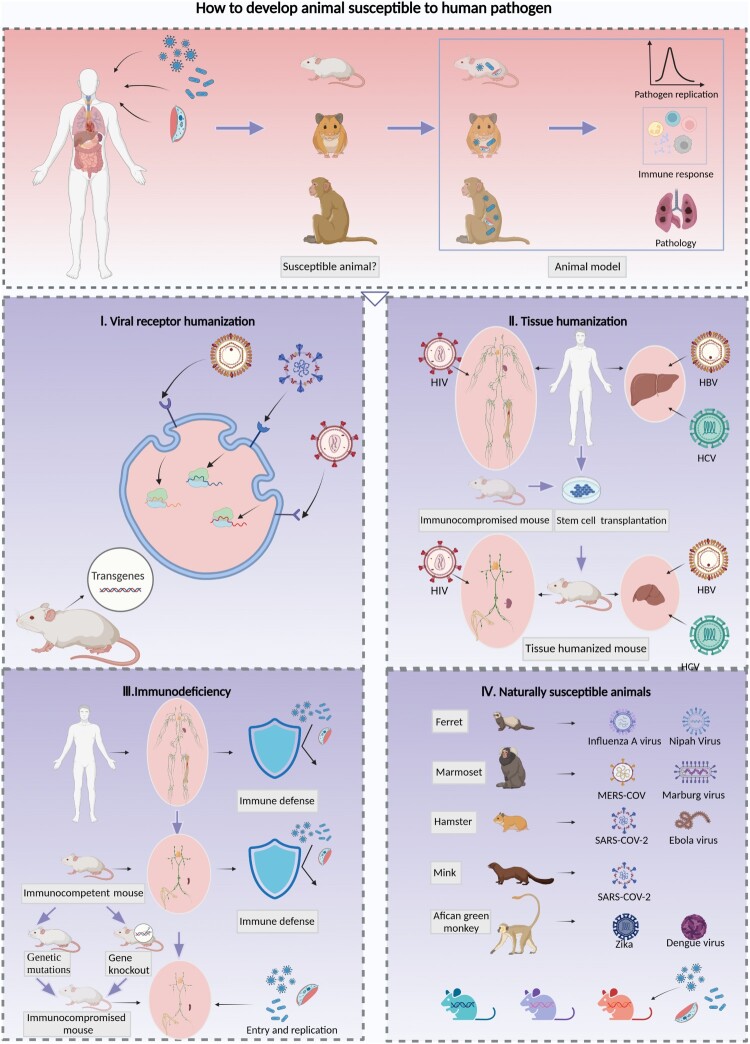
Strategies for the development of animal models with susceptibility to human pathogens. Animal models for human infectious diseases imitate clinically affected patients and reflect the processes of pathogen invasion, replication and shedding as well as the innate and adaptive immune response, tissue injuries and lesions, and clinical symptoms. It is essential to clarify the pathogenesis and develop prophylactic and therapeutic strategies against infection and transmission. The screening of animals that exhibit characteristics similar to those of humans in terms of susceptibility to pathogens is essential and constitutes the first step toward establishing animal models for infectious diseases; four strategies for the development of susceptible animal models were reviewed. I. Expression of human viral receptors via transgenic techniques is a common method for enhancing the susceptibility of rodents to human-origin viruses, which can be achieved by inserting the human receptor gene randomly or in a specific site of the mouse chromosome, and then enhancing the mouse cell susceptibility to human viruses. This method facilitates the development of a viral receptor humanized mouse model for SARS-CoV-2, MERS-CoV and EV71. II. The transplantation of human stem cells or tissues into immunocompromised mice and the generation of immune or liver tissue-humanized animals allows infection by strictly human-tropism-related pathogens such as HIV, HBV and HCV. Furthermore, dual-humanized mice with both hepatocytes and immune cells of human origin could reproduce the HBV or HCV lifecycle in vivo and simulate liver pathogenesis processes of chronic hepatitis B patients, such as inflammation, fibrosis, and ultimately cirrhosis. III. The destruction of immune defenses in immunocompetent animals by genetic mutation or deletion of targeted genes can facilitate pathogenic infection. These animals include the severe immunodeficiency inbred strains Nude, SCID, and NOD/SCID and the genetically modified strains with deletions in antiviral restriction factors related to ISGs or signalling cascade pathways of innate immunity. IV. Some animal species are naturally susceptible to human pathogens; for example, ferrets are susceptible to influenza A virus, marmosets are susceptible to MERS-CoV, and hamsters are susceptible to SARS-CoV-2. In addition, recombinant inbred collaborative cross (CC) mice simulate the diversity of the genetic background and susceptibility to pathogens. Therefore, these mice could be used to identify specific lines susceptible to human pathogen infection.

After an outbreak, scientists must screen or establish animal models susceptible to the pathogens of the emerging infectious disease in a timely manner. Therefore, methods to overcome the limitations posed by a lack of susceptible species or the identification of specific species that are susceptible to human pathogens are reviewed in depth, and these strategies include viral receptor humanization, pathogen-targeted tissue humanization, immunodeficiency induction and screening for naturally susceptible animal species (Figure 1).

### Viral receptor humanization

The genetic diversity of viral receptors across different species narrows the host range for viral tropism. The expression of humanized viral receptors by genetically modified rodents usually enhances the susceptibility of the animals to viruses. Table 1 summarizes the reported receptors for important pathogens that have historically been or currently are the causative agents of epidemics. Among these, some viruses have only one major reported receptor for entry, e.g. dipeptidyl peptidase 4 (DPP4) for MERS-CoV [26] and sodium taurocholate cotransporting polypeptide (NTCP) for HBV [27]. In contrast, some viruses can utilize two or more receptors to initiate the infection process, such as P-selectin glycoprotein ligand-1 (PSGL-1) and scavenger receptor B2 (SCARB2) for human enterovirus 71 (EV71) [28–29]. In addition, some viruses need auxiliary receptors to facilitate entry into the cell. For example, CD4 is the main receptor of HIV, and the coreceptor CCR5 or CXCR4 induces fusion of the viral and cellular membranes [30–31].

**Table 1.** Receptors that potentially enhance the susceptibility of animals to human viruses.

Viruses	Human receptors
Poliovirus	Poliovirus receptor (PVR or CD155)
SARS-CoV	Angiotensin converting enzyme 2 (ACE2)
MERS-CoV	Dipeptidyl peptidase 4 (DPP4)
Zika virus	Neural cell adhesion molecule (NCAM1)
HIV	CD4
HAV	HAVCR1
HBV	Sodium taurocholate cotransporting polypeptide (NTCP)
HCV	CD81
	Scavenger receptor class B type I (SCARBI)
	Claudin-1 (CLDN1)
	Occludin (OCLN)
	Transferrin receptor 1 (TfR1)
	Niemann-Pick C1-like 1 (NPC1L1)
	Epidermal growth factor receptor (EGFR)
	Ephrin receptor A2 (EphA2)
EV71	P-selectin glycoprotein ligand-1 (PSGL-1)
	Scavenger receptor B2 (SCARB2)
SARS-CoV-2	Angiotensin converting enzyme 2 (ACE2)
Influenza A	α-2,6 sialic acid
AIV	α-2,3 sialic acid
CHIKV	Mxra8
RRV	Mxra8
Mayaro virus	Mxra8
ONNV	Mxra8
Ebola virus	Niemann-Pick C1
	Two-pore channels (TPCs)
	T-cell Ig and mucin domain 1 (TIM-1)
Marburg virus	Niemann-Pick C1
	T-cell Ig and mucin domain 1 (TIM-1)
Lassa virus	alpha-dystroglycan (alpha-DG)
	Lysosomal transmembrane protein LAMP1
LCMV	alpha-Dystroglycan (alpha-DG)
Machupo virus	Transferrin receptor 1 (TfR1)
Guanarito virus	Transferrin receptor 1 (TfR1)
Junin virus	Transferrin receptor 1 (TfR1)
Sabia virus	Transferrin receptor 1 (TfR1)
Nipah virus	Ephrin B2

The establishment of a mouse model with viral receptor humanization resolves the immediate problems in the development of animal models for a series of infectious diseases in addition to SARS-CoV-2. For example, the expression of human DPP4 confers susceptibility to MERS-CoV in mice [26]. Compared with wild-type mice, hDPP4 transgenic mice exhibit weight loss, mortality, viral replication in many tissues and bronchointerstitial pneumonia after MERS-CoV infection [32–33]. Animal models of EV71 infection have generally been developed using neonatal mice, which exhibit obvious limitations in vaccine protection evaluation due to the short susceptibility period in neonatal mice. hSCARB2 (an EV71 receptor) transgenic mice show lifelong susceptibility to EV71 and were used to develop an adult model for lethal EV71 challenge, which resolved the bottleneck of evaluating the efficiency of EV71 vaccine candidates [34].

Although the identification of receptors for viruses has facilitated animal model development, the limitations associated with viral receptor humanized mice should be noted. First, the expression of receptors fails to enhance the susceptibility of animals to several viruses, e.g. HBV, HCV and HIV. Further exploration of novel and auxiliary receptors, as well as other host factors that restrict the replication of viruses, might facilitate the permissibility of cells to infection and subsequently enhance the susceptibility of animals to these viruses [35]. Second, compared with those in animals that are naturally susceptible to viruses, the symptoms exhibited by animals with receptor humanization are usually only mild or moderate. For example, compared with hDPP4 mice, marmosets and patients with severe MERS develop moderate to severe disease respiratory disease, bronchointerstitial pneumonia, consolidation in the lungs and changes in blood chemistry indicative of liver or kidney failure [33]. Additionally, differences in the expression quantity and distribution of viral receptors due to different promoters or gene-editing strategies during transgenic mouse model establishment will affect the phenotypes of the resulting animal models. The insertion of hACE2 after knocking out the mouse ACE2 (mACE2) gene and its expression under the promoter of mACE2 usually leads to mild or moderate symptoms in mice infected with SARS-CoV-2 [2,36]. However, transgenic mice expressing hACE2 driven by a cytokeratin 18 promoter (K18) are highly susceptible to SARS-CoV-2, and infection results in a dose-dependent fatal disease course with the specific characteristics of a higher viral titer in the lungs and neuroinvasion [37–38]. These disparities in humanized mouse models can complicate pathogenesis studies and vaccine/drug evaluations.

### Tissue humanization

The use of immunodeficient mice engrafted with human immune cells or hepatocytes, which are designated “humanized mice”, allows productive infection by many human-specific pathogens. Mice bearing mutations in the IL2 receptor common gamma chain combined with either protein kinase DNA-activated catalytic polypeptide mutation or recombination-activating gene mutation were developed in the early 2000s. These mice lack adaptive immunity and exhibit severe deficiencies in innate immunity, and the mice thus fail to reject xenogeneic tissues [39]. When these mice are engrafted with human cells or tissues via the injection of human peripheral blood leukocytes or human CD34^+^ hematopoietic stem cells or subjected to transplantation with human fetal liver or thymus under the kidney capsule and injection of autologous fetal liver hematopoietic stem cells [40], they become susceptible to infection by many human-specific pathogens [41–44].

In addition to nonhuman primate models, humanized mouse models have been successfully developed for HIV. Immunodeficient mice transplanted with human cells or tissues possess most of the major HIV-related host cell types, including CD4^+^ T cells and macrophages, which allow the replication of HIV in vivo and cause CD4^+^ T-cell depletion and other HIV-related immune defects [45–46]. Furthermore, the destruction of immunodeficient mouse hepatocytes permits the engraftment of human hepatocytes and the development of human liver chimeric mice for HBV or HCV infection studies [47–48]. Human liver chimeric mice can become persistently infected with HBV, and a majority of these models have provided valuable virological insights. However, the lack of a functional immune system hinders host immune attack on HBV-targeted hepatocytes and the prevention of chronic hepatitis. Dual-humanized mice altered with both hepatocytes and immune cells of human origin could reproduce the HBV or HCV lifecycle in vivo and simulate liver pathogenesis processes, such as inflammation, fibrosis, and ultimately cirrhosis, as observed in chronic hepatitis B patients [42]. Regarding mycobacterial infection, mice fail to develop granulomas that are common in patients, but immunodeficient mice engrafted with human hematopoietic stem cells form granuloma-like structures after infection in a CD4^+^ T- cell-dependent manner [49]. In addition, humanized mice have also been used as animal models for Nipah virus and dengue virus [50].

The establishment and evolution of humanized mouse models have contributed to a better understanding of human infectious diseases, but the application of humanized mouse models has some limitations. First, suboptimal cell reconstitution and weak responses by human immune components have hindered the reproduction of inflammation and the elucidation of the immune landscape associated with infectious disease [50]. Second, human lymphoid tissue and organ resources are lacking, and human and murine major histocompatibility complex (MHC) molecule presentation and recognition are incompatible; all of these limitations partly affect the immune response [51]. Third, for “multitissue” humanized mouse models, it is critical to avoid potential tissue histoincompatibility when producing a mouse line with a combination of human liver and immune cells; therefore, hematopoietic and hepatic progenitors need to be isolated from the same donor. This can be achieved using human fetal liver tissue; however, there are ethical and legal restrictions associated with this process. Finally, the establishment of humanized models not only depends on the application of complex technologies but also requires advanced technical skills; thus, the standardization and repeatability of such processes need to be improved [52]. Moreover, unlike mice with humanized viral receptors that can be reared and maintained until the emergence of an infectious disease, mouse models with tissue humanization require time for production after emergence of a disease outbreak.

### Immunodeficiency

The replication of pathogens in the tissues or organs of animals is distinct from that in in vitro cultivation because pathogens need to escape host immune surveillance and reach their target tissues. Therefore, it is possible to use rodents that lack certain aspects of the immune system, including broadly immunocompromised mice and specific immune pathway-deficient mice, to enhance the replicative capacity of pathogens in vivo (Table 2).

**Table 2.** Immunodeficient animals that are more susceptible to human pathogens.

Animals	Immunodeficiency	Pathogens
Nude mice	T-cell deficiency	*Rhodococcus equi*
		*Vaccinia viruses*
		*Coccidioides immitis*
		Dengue virus
		Influenza A virus
		Japanese encephalitis virus
		*Yersinia*
		*Leishmania amazonensis*
Nude rat	T-cell deficiency	*Mycobacterium tuberculosis*
		*Toxoplasma gondii*
SCID mice	T- and B-cell deficiency	*Rhodococcus equi*
		*Mycoplasma pulmonis*
		Filaria
		Amebiasis
		*Trypanosoma brucei*
		*Candida albicans*
		Cryptococcosis
		Flavivirus Modoc
		Leishmania
		Hantavirus
NOD/SCID mice	NK-, T- and B-cell deficiency	*Brachylaima cribbi*
		Human enterovirus 71
		*Candida albicans*
NOG/NSG mice	NK-, T- and B-cell deficiency; IL-2 receptor deficiency	*Babesia microti*
		*Strongyloides stercoralis*
		Epstein–Barr virus
		*Demodex musculi*
		HTLV-1^a^
		HBV
CBA/N mice	B-cell deficiency	*Mycoplasma pulmonis*
		*Cryptococcus neoformans*
C3H/HeN mice	Interferon deficiency	Group A *streptococci*
Beige mice	NK-cell deficiency	*Leishmania*
		*Salmonella choleraesuis*
		*Candida albicans*
		Cryptococcosis
		*Mycobacterium avium*
Rag2^−/−^ mice	T- and B-cell function deficiency	HSV-1^b^
		Reovirus
IFNAR^−/−^ mice	Type-I interferon function deficiency	Zika virus
		Rift Valley fever virus
		Ebola virus
		Yellow fever virus
		SARS-CoV-2
		AHFV^c^
		West Nile virus
		Dengue virus
		Japanese encephalitis virus
		Sudan virus
		Reston virus
		Tai Forest virus
		Marburg virus
		Ravn virus
		Lassa virus
		CCHFV^d^
		SFTSV^e^
		Hendra virus
		Nipah virus
		VEEV^f^
		Chikungunya virus
		*Salmonella Typhimurium*
		*Trypanosoma cruzi*
		Rabies virus
		*Mycobacterium tuberculosis*
		*Pseudorabies virus*
		Echovirus
		Reovirus
STAT-1^−/−^	Type I interferon pathway deficiency	Ebola virus
		LCMV^g^
		Herpes simplex virus
		Dengue virus
		Lassa virus
		*Cryptococcus neoformans*
		*Mycobacterium tuberculosis*
Grail^−/−^	Reduced T-cell responsiveness	Influenza A virus
TLR2^−/−^	Toll-like receptor deficiency	*Staphylococcus aureus*
		*Borrelia burgdorferi*
		*Streptococcus pneumoniae*
		*Mycobacterium tuberculosis*
		*Borrelia burgdorferi*
		*Candida albicans*
MyD88^−/−^	TLR/IL-1 receptor family signalling deficiency	*Mycobacterium tuberculosis*
		*Staphylococcus aureus*
		*Clostridium difficile*

Note: a, human T-cell leukemia virus type 1; b, herpes simplex virus type 1; c, Alkhurma hemorrhagic fever virus; d, Crimean-Congo hemorrhagic fever virus; e, severe fever with thrombocytopenia syndrome virus; f, Venezuelan equine encephalitis virus; g, lymphocytic choriomeningitis virus.

Three scenarios are usually associated with enhancement of the susceptibility of animals to pathogens via the immunodeficiency strategy. First, the replication capability and pathogen burden are enhanced. For example, after infection, the number of *Leishmania major* parasites in SCID mice is 100-fold higher than that in BALB/c mice [53]. Second, the pathogenicity is enhanced after infection. For example, compared with immunocompetent mice that survive hantavirus infection, SCID mice inoculated with hantavirus die 32–35 days after infection [54]. Third, the susceptibility of mice of a specific sex and age is enhanced. For example, the infection of C57BL/6J mice with *Brachylaima cribbi* is sustained for only 9–12 weeks, and mature male or adolescent female mice are more susceptible to infection, as demonstrated by reductions in the worm burden, fecundity and egg load [55].

The function of the IFNsystem is to detect pathogen invasion and trigger a response that limits the replication and spread of the pathogen. Type I IFNs perform antiviral functions in vivo by activating and regulating cells of both the innate and adaptive immune compartments. IFN-I receptors play essential roles in IFN-I-mediated signal transduction [56–57]. Mice lacking the IFN-I receptor (*Ifnar^−/−^*) were developed in 1994 and are unresponsive to the effects of IFN-Is. These knockout (KO) mice exhibit enhanced susceptibility to a broad range of viruses, such as vesicular stomatitis virus, Semliki Forest virus, vaccinia virus and lymphocytic choriomeningitis virus, as shown by an elevated viral burden and high pathogenicity [56,58–62].

Signal transducer and activator of transcription 1 (STAT1) is a critical component of the IFN-I signalling pathway. It is activated by the binding of IFN-Is with IFN-α/β receptor 1 and 2 subunits and subsequent formation of a trimolecular complex to regulate the expression of a series of IFN-regulated genes orchestrating the host antiviral response. The essential role of STAT1 in the IFNγ pathway has been demonstrated by the enhanced susceptibility of STAT1*^−^*^/*−*^ mice to infection with mouse cytomegalovirus, *Lassa virus* and *Listeria monocytogenes* [63–68].

Members of the Toll-like-receptor (TLR) family, which are receptors for invasive pathogen recognition, initiate immune signalling, orchestrate inflammatory responses, and trigger a specific adaptive immune response. Disruption of the TLR2 gene significantly decreases the survival of mice infected with *Staphylococcus aureus, Streptococcus pneumoniae* and *Mycobacterium tuberculosis* [69–71]. Similarly, TLR7 deficiency in mice increases the viral load in the airway epithelium and results in cardinal pathophysiologic features of asthma upon inoculation with respiratory tract pathogens, such as a pneumonia-causing virus [72].

Most TLRs and interleukin-1 receptors (IL-1Rs) transmit signals via myeloid differentiation primary response protein 88 (MyD88) to recruit interleukin-1 receptor-associated kinase 4 (IRAK-4) and activate multiple transcription factors. MyD88-deficient mice are susceptible to more than 45 pathogens, including 27 bacteria, 7 protozoa, 8 viruses and 4 fungi, and these pathogens include some important representative pathogens, such as *S. aureus*, *S. pneumoniae*, *Haemophilus influenzae*, *Salmonella typhimurium*, *M. tuberculosis*, SARS-CoV and rabies virus, which have been well reviewed [73].

### Naturally susceptible animals

Immunocompetent animals that are susceptible to specific pathogens could not only demonstrate the process of viral replication and pathological changes but also illustrate the full landscape of the immune response against pathogen invasion; therefore, these animals are invaluable for immunology and pathology studies. Fortunately, certain laboratory and farm animals have been found to be susceptible to specific human pathogens (Table 3). Although some commonly used laboratory animals show a broad spectrum of sensitivity to human pathogens, these animals were not included in this study due to their wide availability.

**Table 3.** Specific animals that are susceptible to human pathogens.

Animal	Pathogen
Guinea pig	*Mycobacterium tuberculosis*
	Junín virus
	Influenza virus
	*Treponema pallidum*
	Respiratory syncytial virus
	*Streptococcus pneumoniae*
Syrian hamster	SARS-CoV-2
	Nipah virus
	Hendra virus
	West Nile virus
	Ebola virus
	Marburg virus
	Rift Valley fever virus
	SARS-CoV
	Prions
	Yellow fever virus
	Influenza A virus
	*Clostridium difficile*
	*Helicobacter*
	*Leishmania*
	*Babesia*
Marmoset	MERS-CoV
	*Coxiella burnetii*
	Influenza A virus
	Marburg virus
	GB virus B
	Yellow fever virus
	Herpes simplex virus 1
	*Francisella tularensis*
	Zika virus
	*Mycobacterium tuberculosis*
Ferret	Influenza A virus
	SARS-CoV-2
	SARS-CoV
	Nipah virus
	Hendra virus
	Respiratory syncytial virus
Woodchuck	Woodchuck hepatitis virus^a^
Mongolian gerbil	*Helicobacter pylori*
	Hepatitis E virus
Naked mole rat	Herpes simplex virus type 1
African green monkey	Nipah virus
	Zika virus
	Simian immunodeficiency virus^b^
	SARS-CoV-2
	MERS-CoV
	Respiratory syncytial virus
	Dengue virus
	Machupo virus
	*Yersinia pestis*
	*Leishmania*
	Anthrax
Lemur	*Hymenolepis nana*
	*Encephalitozoon intestinalis*
	*Yersinia pseudotuberculosis*
Baboon	Cryptosporidiosis
	Pertussis
	Ebola virus
	Zika virus
	*Plasmodium knowlesi*
Vole	SARS-CoV
	Puumala virus
	Prions
Chinese hamster	SARS-CoV-2
	SARS-CoV
Civet	SARS-CoV
	Rotavirus
	Lyssavirus
Mink	*Staphylococcus aureus*
	Hepatitis E virus
	Influenza A virus
	SARS-CoV-2
	*Streptococcus phocae*
Tree shrew	SARS-CoV-2
	Kaposi’s sarcoma-associated herpesvirus
	Epstein–Barr virus
	Hepatitis B virus
	Hepatitis C virus
	Hepatitis E virus
	*Mycobacterium tuberculosis*
	Zika virus
	Influenza A virus
	Herpes simplex virus
	Dengue virus
Collaborative cross or diversity outbred mice	*Aspergillus fumigatus*
	*Klebsiella pneumoniae*
	*Pseudomonas aeruginosa*
	Ebola virus
	SARS-CoV
	SARS-CoV-2
	Zika virus
	West Nile virus
	*Mycobacterium tuberculosis*

Note: a, surrogate animal model for HBV infection; b, simulated AIDS.

The Syrian hamster is an extraordinarily effective animal model for human infectious diseases. More than 70 different pathogens have been studied using the Syrian hamster model, and these include viruses such as West Nile virus, Nipah virus, Ebola virus, Marburg virus, SARS-CoV and SARS-CoV-2 and pathogenic bacteria or parasites, such as *Leptospira interrogans*, *Clostridioides difficile*, *Leishmania donovani* and *Schistosoma haematobium* [74–75]*.* Furthermore, Syrian hamsters exhibit high sensitivity to SARS-CoV-2 infection and well simulate the viral replication, transmission and pathological features of SARS-CoV-2 infection in human patients [76]. The mechanism of the susceptibility of Syrian hamsters to a broad spectrum of human-specific pathogens remains to be elucidated, and previous studies have suggested that the similarity of viral receptors between Syrian hamsters and humans plays a vital role in susceptibility. For example, the high similarity between ACE2 of Syrian hamster and human amino acid residues binding with receptor-binding domains (RBDs) enhances the binding activity of ACE2 of Syrian hamster to SARS-CoV-2 [77]. In addition, similar to ferrets and humans, Syrian hamsters have appreciable amounts of SAα2,6Gal in the distal end of their nasal turbinates and SAα2,3Gal in their lungs, which make them susceptible to human influenza virus [78].

In addition to rhesus monkeys, other less frequently used nonhuman primates, such as African green monkeys, baboons and marmosets, have gradually been found to be sensitive to infection with specific human pathogens and have advantages in some research areas [79–81]. For example, similar to chimpanzees and mangabey, the African green monkey is one of the natural hosts of simian immunodeficiency virus (SIV), but its infection does not progress to AIDS; thus, this animal is an excellent model for studying the mechanisms involved in controlling disease progression [82]. In addition, upon challenge with SARS-CoV-2, African green monkeys exhibit variably severe histopathologic changes typical of coronavirus respiratory disease, which are characterized by interstitial pneumonia [83]. Compared with conventional nonhuman primates, the common marmoset (*Callithrix jacchus*), a small New World primate, has become an extensively used model in infectious disease research [84]. Marmosets have an invaluable benefit in MERS-CoV research. Compared with rhesus macaques, which develop mild to moderate respiratory disease similar to mild MERS cases, marmosets develop moderate to severe respiratory disease, which simulates severe disease in the clinic [33]. Furthermore, marmosets have high susceptibility to influenza A virus, Zika virus (ZIKV), Marburg virus, monkeypox virus and yellow fever virus [85–87].

Animals that are naturally susceptible to specific human pathogens provide ideal models for studying infectious diseases; however, the lack of accessibility due to the small number of species and the lack of gene-editing technologies and reagents, particularly antibodies for these animals, hinders their widespread use as models [74].

In addition to interspecies variations, genetic polymorphism within a single animal species contributes to disparities in susceptibility, leading to variation in the host response to an infectious pathogen between individuals. This variation can be defined based on the immune response strength, and the disease severity ranges from asymptomatic to mild, moderate, severe or lethal. Host susceptibility to pathogens is considered a complex trait that is controlled by gene–environment interactions, resulting in extensive phenotypic variations between individuals. To mimic the genetic diversity of humans, a unique mouse model termed a collaborative cross (CC) line was developed via full-reciprocal intercrosses of eight founder mouse strains to generate inbred recombinant CC mouse model lines. Due to the inclusion of three wild mouse strains among the eight founder mouse strains, the CC lines show particularly high diversity in disease phenotypes caused by polymorphisms [88]. Notably, a series of CC lines have been screened and defined as susceptible to specific viral pathogens, including Ebola virus, SARS-CoV, SARS-CoV-2, ZIKV and West Nile virus, as well as bacterial pathogens, such as *K. pneumoniae, Pseudomonas aeruginosa* and *M. tuberculosis* [89–92]. To date, extensive genetic studies of infectious diseases have been successfully performed using the CC mouse model, and these studies mainly focused on the identification of candidate genes underlying the quantitative trait loci (QTLs) for elucidating the genetic basis of host susceptibility to various pathogens. For example, CC lines show significant differences in the mean survival time after infection with *K. pneumoniae*. The QTL mapping of phenotypes indicated that susceptibility to *K. pneumoniae* is a complex trait controlled by at least three loci, *Kprl1*, *Kprl2* and *Kprl3*. A merged analysis by imputation and testing the association of sequence variants, with segregation based on inferring the alleles of each CC line depending on its genome mosaic for single nucleotide polymorphisms (SNPs) and sequence variation among the founder strains, predicted several genes that are potentially related to susceptibility to *K. pneumoniae*, including *Ikbkap*, *Actl7a*, *Actl7b*, *Ctnnal1*, *Bag4*, *Pik3c3*, *Rit2*, *Slc25a46*, *Sap130* and *Pik3c3*. These results shed light on the immune mechanisms and pathogenesis of *K. pneumoniae* infection and can guide the development of targeted drugs with a focus on susceptibility-related genes [92].

## Perspectives

Exhaustively listing all the known animal species and strains that are susceptible to the whole spectrum of identified human pathogens is beyond the scope of the present review. However, from the perspective of enhancing the susceptibility of animal species to human pathogens of concern, we summarized four tools or methods for the modification or screening of specific animals for model development: viral receptor humanization, pathogen-targeted tissue humanization, immunodeficiency induction, and screening of susceptible animal species. In addition, other tools or methods should be noted, and these include the use of germ-free mice to enhance susceptibility to infection and the induction of comorbidities in animals to aggravate the severity of disease after infection. Many studies support the notion that the gut microbiota plays a critical role in the process of pathogens invading the host intestine, and the gut microbiota protects animals against pathogenic bacterial infection via competitive metabolic interactions, localization to intestinal niches and induction of host immune responses [93–94]. Moreover, comorbidity will aggravate the illness upon infection. For COVID-19, although in the susceptibility of humans and animals to SARS-CoV-2 did not differ among age, sex and health status, patients or animals with basic diseases such as hypertension, diabetes, chronic obstructive pulmonary disease, cardiovascular diseases, cancer and weak health status related to older age exhibit more severe diseases [95–97]. Organoids, which are derived from stem cells or adult tissues in a specific three-dimensional microenvironment, have recently been shown to mimic the characteristics of organs in vivo. Organoids provide a completely humanized model for infectious diseases such as ZIKV and COVID-19, which overcomes the restriction on the susceptibility of animals to human pathogens [98–99]. However, as a subrogate of animal models, organoids need to be improved in depth to resolve the limitations due to the lack of vasculature, immune cells, and interorgan communication [100].

Notably, a certain amount of time is needed to establish susceptible animal models for a novel pathogen, but this duration is unacceptable in emergency outbreaks. Therefore, we propose the development of animals that are susceptible to human pathogens that have caused important infectious diseases or infection with what will result in severe diseases in advance via techniques including but not limited to the four tools or methods described above. We hypothesize that this approach will produce a series of susceptible animal resources and decrease the time required for animal model development when epidemics occur. Then, based on the animal resources described above, the pathogen susceptibility spectrum of each animal species or strain should be systematically tested and screened first. These data, which include information on pathogen replication, host immune response, tissue pathological changes and symptoms of each animal species or strain to specific pathogens, will greatly facilitate the rapid and accurate selection and subsequent use of animals to develop models for emerging infectious diseases. Third, it is worth comparing similarities and differences in phenotypes, including pathogen replication and distribution, transmission, immune response, pathological injury and symptoms, among different animal models and patients. These results will notably enhance the precise application of animal models in biomedical research. Finally, the development of gene-editing tools and antibodies for marmosets, African green monkeys, ferrets, and Syrian hamsters is important for facilitating their timely and accurate application in studies of emerging infectious diseases [74]. In addition, in responses to outbreaks of emerging infectious diseases with novel pathogens, it is necessary to test the susceptibility against novel pathogens using animals that are susceptible to existing pathogens adjoined to the novel pathogen, a strategy that has been used to select hACE2 mice (susceptible to SARS-CoV) and thus develop an animal model for COVID-19. The screening of susceptible animals for novel pathogens from well-established animal resources with a well-known susceptibility spectrum will increase the chance of obtaining suitable animals for model development and save time in the development of susceptible animals from scratch.
